# Drug-associated cues and drug dosage contribute to increased opioid seeking after abstinence

**DOI:** 10.1038/s41598-021-94214-4

**Published:** 2021-07-21

**Authors:** Mary Tresa Zanda, Gabriele Floris, Stephanie E. Daws

**Affiliations:** 1 Center for Substance Abuse Research, Temple University, 3500 N Broad St, MERB/ Rm 847, Philadelphia, PA 19140 USA; 2 Department of Neural Sciences, Temple University, Philadelphia, PA USA

**Keywords:** Reward, Addiction

## Abstract

Patients with opioid use disorder experience high rates of relapse during recovery, despite successful completion of rehabilitation programs. A key factor contributing to this problem is the long-lasting nature of drug-seeking behavior associated with opioid use. We modeled this behavior in a rat drug self-administration paradigm in which drug-seeking is higher after extended abstinence than during the acute abstinence phase. The goal of this study was to determine the contribution of discrete or discriminative drug cues and drug dosage to time-dependent increases in drug-seeking. We examined heroin-seeking after 2 or 21 days of abstinence from two different self-administration cue-context environments using high or low doses of heroin and matched animals for their drug intake history. When lower dosages of heroin are used in discriminative or discrete cue protocols, drug intake history contributed to drug-seeking after abstinence, regardless of abstinence length. Incubation of opioid craving at higher dosages paired with discrete drug cues was not dependent on drug intake. Thus, interactions between drug cues and drug dosage uniquely determined conditions permissible for incubation of heroin craving. Understanding factors that contribute to long-lasting opioid-seeking can provide essential insight into environmental stimuli and drug-taking patterns that promote relapse after periods of successful abstinence.

## Introduction

The prevalence of opioid use disorder (OUD) and overdose deaths involving opioids has drastically increased in the past 2 decades^1^. In the United States, nearly 130 people die of opioid overdose every day and approximately $78 billion will be spent annually treating opioid use disorder^2,3^. More than 80% of OUD patients relapse within the first year^4,5^. For many individuals, drug craving persists over time and stable recovery is not attained until nearly 5 years after the last drug exposure^5^. Understanding the neurobiology that contributes to progressive and perseverant drug-seeking is critical for the expansion of strategies to treat relapse in patients suffering from OUD.

Variability in animal, as well as human, behavior is inevitable and finding conditions that contribute to high or low levels of drug-seeking may reveal important insights into treating different populations of OUD patients. For many drug classes, drug-seeking increases during abstinence, growing stronger over time for individuals recovering from substance use disorder^6–10^. This phenomenon is referred to as the ‘incubation of drug craving’ and can be modeled successfully in rodents for many drug types, including opioids^11^. Time-dependent increases in seeking for the highly addictive opioid heroin have been well-described in the literature^12^. Initially, continuous long-access exposure to opioids (e.g. 9 hours [h] per day) was employed to induce incubation of heroin craving^12^. More recently, incubation for heroin seeking has been reported in rat models after continuous daily 6 h and even 3 h sessions using operant conditioning^13–16^. This translates to human drug-seeking patterns in which humans may be addicted to heroin but are not continuously administering it while they are awake^17^.

Rat self-administration paradigms described in the literature that elicit incubation of heroin craving typically use discrete cues in conjunction with drug infusion, leading to the formation of a learned drug-cue association. Discrete cues are neutral stimuli, such as a light or tone, that become conditioned reinforcers when paired with a drug infusion during a self-administration protocol. These cues are then presented in the absence of the drug during ‘relapse’ tests to assess the incubation of heroin craving^18^. However, discriminative cues are also present prior to drug-seeking, as they signal for drug availability, and represent a critical target for therapeutic development. In rat self-administration protocols, discriminative drug cues may also be light conditions or tones but importantly, the cue is not temporally paired with a drug infusion. Instead, discriminative drug cues are environmental stimuli that function as ‘occasion setters’ to indicate whether the conditions are permissible for engaging in drug taking^19^. While the contribution of discrete and discriminative cues to incubation of cocaine craving has recently been described, the contribution of the two types of cues to the development of time-dependent heroin seeking has not been examined. In addition, incubation behaviors have not been studied in low-dose (less than 0.05 mg/kg/infusion) heroin self-administration paradigms. Yet, heroin is used in a range of doses by humans and moreover, heroin acquisition in rat self-administration paradigms is supported at lower doses^20–22^. The goal of this study was to determine the contribution of drug-associated cues and drug dosage to the incubation of heroin craving. After successfully modeling time-dependent increases in heroin seeking utilizing a well-established incubation protocol that paired discrete visual and auditory cues with the drug infusion, we assessed the relapse behavior in animals that self-administered lower dosages of heroin in the same context and when cues were manipulated as discriminative stimuli signaling drug availability. Our data demonstrated that incubation in rat heroin self-administration paradigms was shaped by drug dose and drug cues. Distinct interactions between drug dosage and drug cues determined whether drug seeking remained steady throughout abstinence or increased over time. These findings provide insight into the behavioral mechanisms of the incubation of heroin craving, suggesting that not all learned experiences are able to trigger exacerbation of long-lasting drug-seeking behavior. Similarly, opioid users may process drug seeking cues differently depending on the extent of their drug usage.

## Results

### Experiment 1: Heroin seeking increased after extended abstinence from higher infusion doses

**Figure 1:**
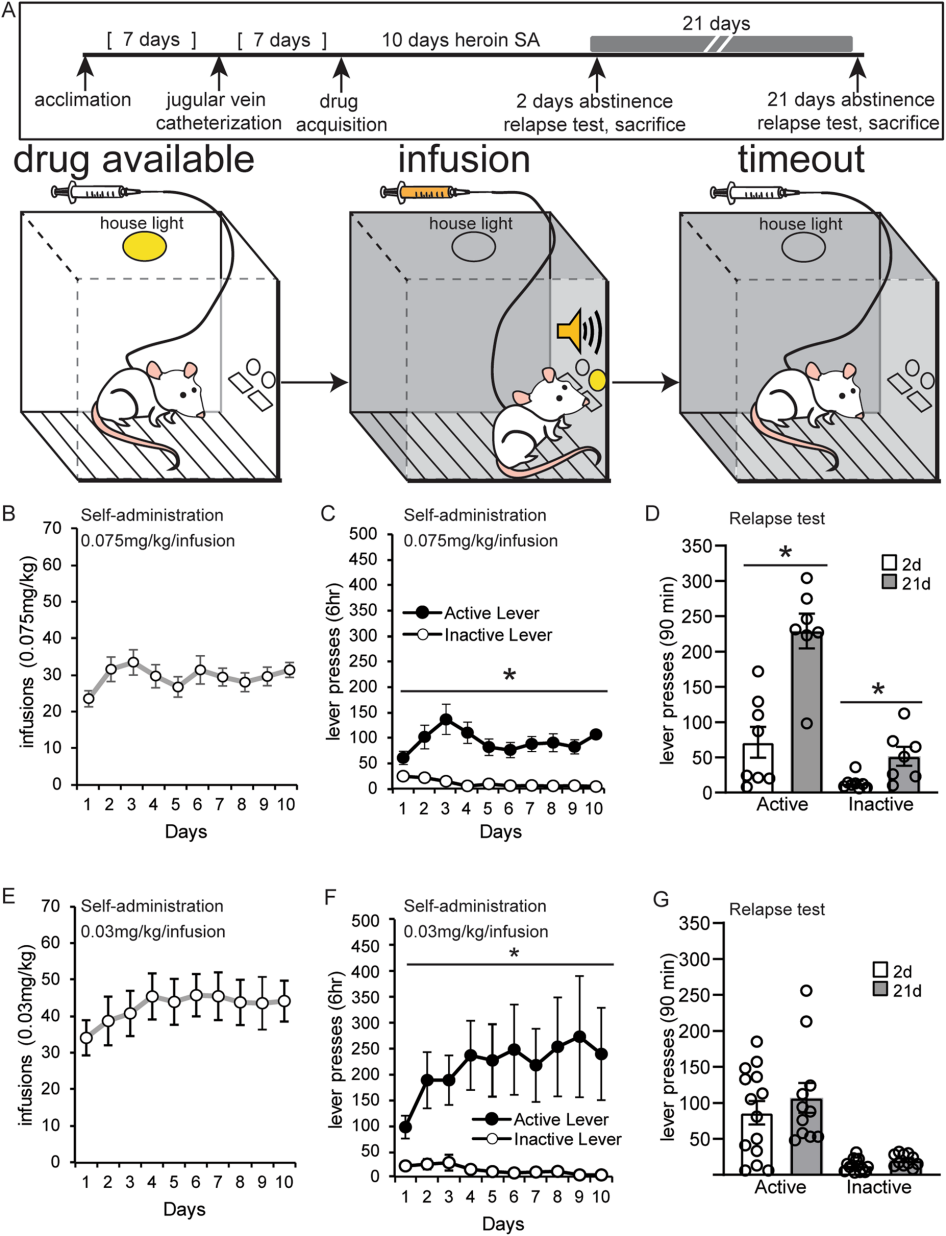
Establishment of a rat incubation of opioid craving protocol with discrete drug cues. (**A**) An overview of the protocol to measure drug seeking after abstinence from self-administration of heroin paired with discrete drug cues. Animals underwent surgery for jugular vein catheterization then acquired heroin self-administration in 6-h daily sessions for 10 days. A relapse test was performed after 2 or 21 days of abstinence from heroin. During acquisition, the house light signaled the availability of the drug. Active lever pressing resulted in illumination of a light above the active lever and an auditory cue during drug infusions. A 20 second (s) timeout period followed each infusion, in which no lights were illuminated in the chamber. (**B**, **C**, **E**, **F**) The number of infusions and lever presses during heroin self-administration at a 0.075 mg/kg/infusion dosage (**B**, **C**) and a lower 0.03 mg/kg/infusion dosage (**E**, **F**). Error ± standard error of the mean (SEM). (**D**, **G**) Lever pressing during the relapse test after forced abstinence from the 0.075 mg/kg/infusion dosage (**D**) and the 0.03 mg/kg/infusion dosage (**G**). *p < 0.05.

Incubation of heroin seeking has been demonstrated in many labs using modified experimental settings, including a range of doses from 0.05 to 0.1 mg/kg/infusion, 3–9 h sessions and various combinations of visual cues with tones^12,13,16,23,24^. We established a well-described paradigm of incubation of opioid craving in our laboratory using a rat model of heroin self-administration and a standard dose of 0.075 mg/kg heroin per infusion^16,24–26^ (Fig. 1B–D). This protocol utilized both auditory and visual discrete cues during drug infusion and resulted in stable self-administration of heroin in 6-h daily sessions over 10 days (Fig. 1B, C). Rats quickly developed a significant preference for the active lever over the inactive lever that was maintained for the duration of acquisition. A significant main effect of lever and a time × lever interaction were observed (two-way repeated measures ANOVA, main effect of lever: *F*(1,30) = 30.23; *p* < 0.0001; time × lever interaction: *F*(9,270) = 3.04; *p* = 0.0018; Fig. 1C). After acquisition, rats were returned to their home cage for either 2 or 21 days of forced abstinence. To assess time-dependent changes in drug-seeking after forced abstinence, rats were placed back in the self-administration chambers for a 90-min relapse test. All the contextual, visual and auditory cues present during self-administration were available during the relapse test, but active lever pressing did not result in drug infusions. As previously described in the literature, extended abstinence from the 0.075 mg/kg/infusion heroin dose resulted in increased drug seeking during the relapse test (unpaired *t* test of 2 vs. 21 day relapse, active lever: *t*(13) = 4.827; *p* = 0.0003; inactive lever: *t*(13) = 2.932; *p* = 0.012; Fig. 1D).

We next examined the robustness of opioid incubation after a low-dose protocol, below the threshold of what has previously been described. In a separate group of animals, rats self-administered heroin in 6-h daily sessions at a dose of 0.03 mg/kg/infusion under the same experimental conditions (Fig. 1A, E–F). Rats easily discriminated the active versus inactive lever and a significant main effect of lever was observed over the course of the 10 day protocol but not a time × lever interaction (two-way repeated measures ANOVA, main effect of lever: *F*(1,48) = 9.44; *p* = 0.0035; time × lever interaction: *F*(9,432) = 1.767; *p* = 0.0725) (Fig. 1F). However, the 90-min relapse test revealed no significant differences in active lever presses at 2 versus 21 days of abstinence from the lower 0.03 mg/kg/infusion heroin dosage (Fig. 1G). While we did not detect incubation behavior with this low dose, it is worth noting that animals displayed perseverative behavior after 21 days of abstinence and had significantly more active lever presses at the 60–90 min time block of the relapse test than animals that underwent 2 days of abstinence (2 day mean = 4.77 vs. 21 day mean = 20.63; *p* < 0.001; Supplemental Fig. 2). Thus, incubation of heroin craving from discrete drug cues was not observed at the 0.03 mg/kg infusion dosage.

### Experiment 2: Discriminative drug cues elicited steady heroin seeking after extended abstinence

**Figure 2:**
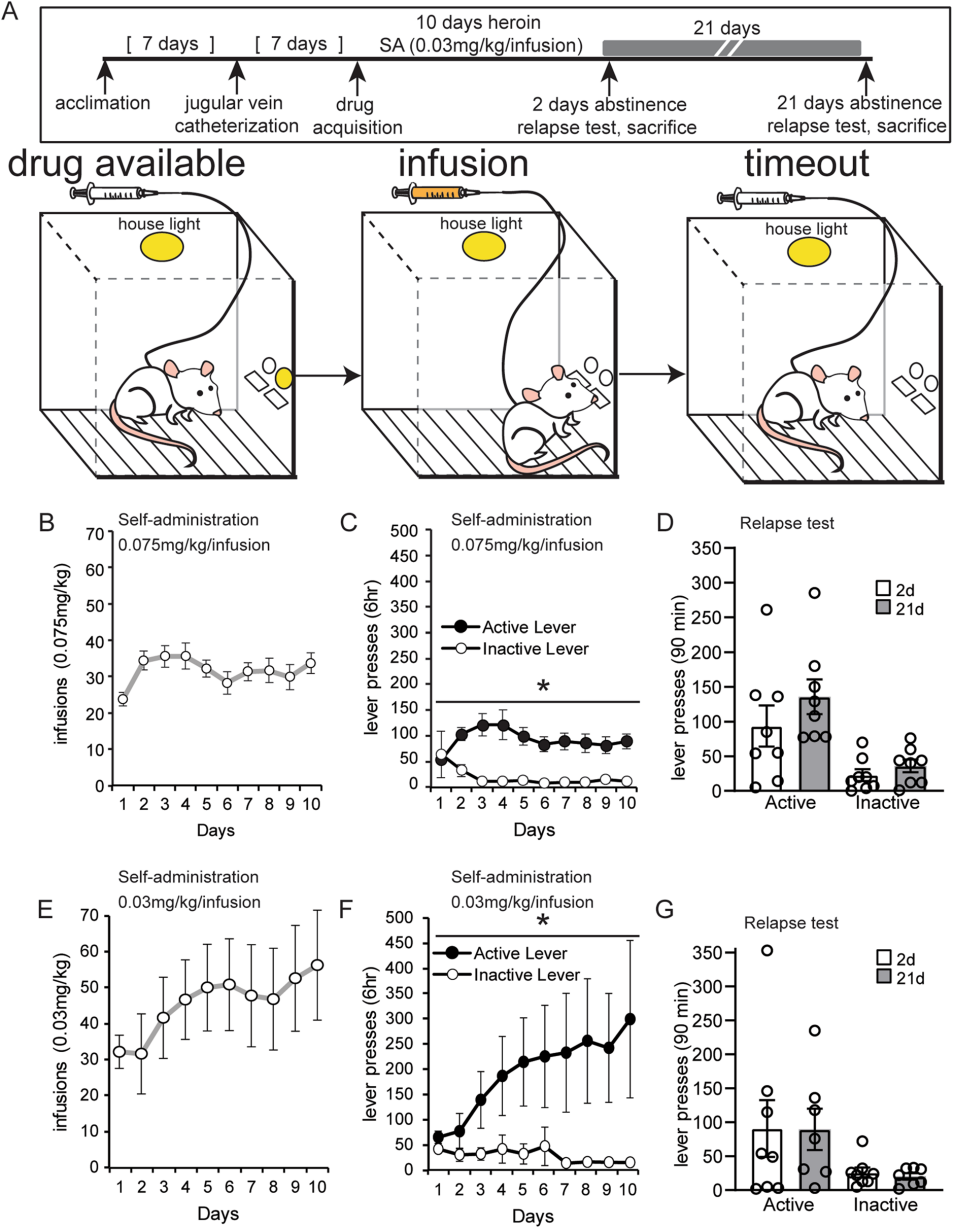
Discriminative cues produce steady drug-seeking during abstinence. (**A**) An overview of the protocol to measure drug seeking after abstinence from heroin self-administration using discriminative drug cues. Animals underwent an identical experimental protocol as described in Experiment 1 except with modified light and tone cues. (**B**, **C**, **E**, **F**) The number of infusions and lever presses during heroin self-administration at a 0.075 mg/kg/infusion dosage (**B**, **C**) and a lower 0.03 mg/kg/infusion dosage (**E**, **F**). (**D**, **G**) Lever pressing during the relapse test after forced abstinence from the 0.075 mg/kg/infusion dosage (**D**) and the 0.03 mg/kg/infusion dosage (**G**). Error ± SEM. *p < 0.05.

We examined the requirement of specific paired cues for the establishment of the incubation of heroin seeking behavior by manipulating the cue light, using it as a discriminative cue for drug availability instead of pairing it with drug infusion (Fig. 2A). In experiment 2, rats self-administered 0.075 mg/kg/infusion or 0.03 mg/kg/infusion in 6-h daily sessions but the house light remained on during the session (Fig. 2A). Drug availability was denoted by the presence of a light above the active lever and active lever pressing resulted in infusion of heroin but no tone cue. A significant main effect of lever and a time × lever interaction were observed when the 0.075 mg/kg dosage was used in experiment 2 (two-way repeated measures ANOVA, main effect of lever: *F*(1,30) = 23.48; *p* < 0.0001; time × lever interaction: *F*(9,270) = 3.34; *p* = 0.0007) (Fig. 2C). When animals were presented with discriminative drug cues after abstinence from the high dosage, incubation was not observed (unpaired *t* test of 2 vs. 21 day relapse, active lever at 0.075 mg/kg/infusion dosage: *t*(14) = 1.088; *p* = 0.2948; Fig. 2D).

When the lower 0.03 mg/kg/infusion dosage of heroin was used in the discriminative drug cue protocol during experiment 2, we observed a significant time × lever interaction but no main effect of lever (two-way repeated measure ANOVA: *F*(9,270) = 2.766; *p* = 0.0041; Fig. 2F). Similar to the 0.075 mg/kg/infusion heroin dose, incubation of craving was not observed after 21 days of abstinence from the lower 0.03 mg/kg/infusion dosage as drug seeking remained steady at both time points tested (unpaired *t* test of 2 vs. 21 day relapse, active lever at 0.03 mg/kg/infusion dosage: *t*(13) = 0.027; *p* = 0.979; Fig. 2G).

### Lack of incubation with low heroin doses and discriminative cues cannot be attributed to differences in learning

Using published criterion that acquisition is achieved if the animal makes at least 10 responses on the active lever and has at least a 2:1 ratio in active: inactive lever presses for the last three consecutive sessions, we measured the number of rats that acquired self-administration across the two experiments at two heroin doses^21,27^. When the 0.075 mg/kg/infusion dosage was used with discrete drug cues in experiment 1, 100% of rats acquired self-administration (Table 1). Reducing the dosage to 0.03 mg/kg/infusion lowered acquisition to 96%. Using only discriminative drug cues in experiment 2 further reduced acquisition to 93.8% for the 0.075 mg/kg/infusion dose and 75% for the lower 0.03 mg/kg/infusion dose.

**Table 1:** Descriptive statistics for animals that met criteria of two-fold more lever pressing on the active versus inactive lever.

	% Meeting criteria	Active lever presses (last 3 days)			Inactive lever presses (last 3 days)			Infusions (last 3 days)		
		Mean	Max	Min	Mean	Max	Min	Mean	Max	Min
**Experiment 1: discrete drug cues**										
0.075 mg/kg heroin dose-discrete drug cues	100 (16/16)	92.2	260.3	19.7	4.8	14.0	0.3	29.6	47.0	16.0
0.03 mg/kg heroin dose-discrete drug cues	96 (24/25)	265.8	2303.0	24.3	9.3	53.7	1.0	45.3	152.0	16.0
**Experiment 2: discriminative drug cues**										
0.075 mg/kg heroin dose-discriminative drug cues	93.8 (15/16)	86.4	274.0	21.0	9.4	26.0	2.0	32.4	58.0	15.0
0.03 mg/kg heroin dose-discriminative drug cues	75 (12/16)	369.2	2335.0	7.7	8.6	28.3	1.7	66.3	215.7	6.7

To rule out differences in incubation that may be attributed to inadequate acquisition, we compared the acquisition and relapse behavior of animals from experiments 1 and 2 that met the acquisition criteria. Animals in experiment 1 self-administered a higher number of infusions at the 0.03 mg/kg heroin dosage than their higher dosage counterparts and overall were more variable. The range of infusions over the last 3 days of self-administration in animals that met acquisition criteria was 16–152 with the 0.03 mg/kg heroin dosage, while animals had an infusion range of 16–47 with the higher 0.075 mg/kg heroin dose under the same protocol (Table 1). After the initial day, rats that acquired self-administration stabilized to an average of ~ 45 infusions during the last 3 days of self-administration, versus the ~ 30 infusions found with the high dose (Table 1). In experiment 2, the range of infusions over the last three days of self-administration in animals that met acquisition criteria was 15–58 with the 0.075 mg/kg heroin dosage. On the contrary, even if most rats acquired self-administration of heroin with the 0.03 mg/kg heroin dosage and discriminative cues, a large amount of variability was observed in the number of infusions made by animals that met acquisition criteria, ranging from 6.7 to 215.7 during the last 3 days of self-administration (Table 1). We reanalyzed the relapse behavior of animals from experiments 1 and 2, excluding animals that did not meet the acquisition criteria of two-fold more active lever presses than inactive lever presses. Importantly, similar levels of active lever pressing were observed as reported above in Figs. 1D, G and 2D, G (Supplemental Fig. 3).

**Figure 3:**
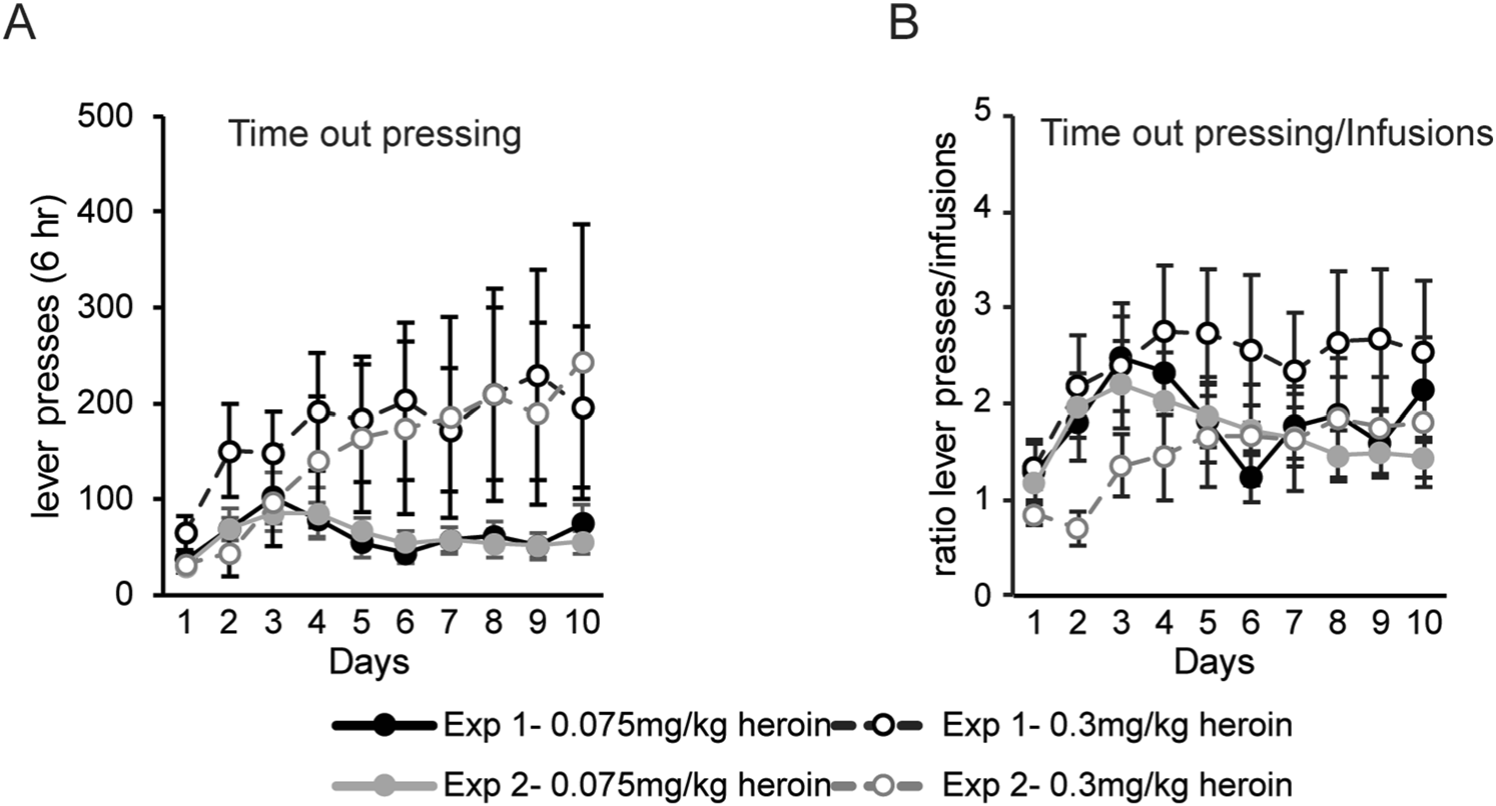
The amount of time-out pressing was not changed by drug dosage or drug-associated cues. (**A**) The number of lever presses on the active lever during the 20-s time-out period that followed each infusion was measured for each experimental protocol. (**B**) The amount of time-out pressing was calculated by adjusting for the total number of infusions made in each experimental protocol. Shown are active lever time-out presses divided by the number of infusions for experiments 1 and 2. Error ± SEM.

As mentioned above, a high amount of variability was observed with the 0.03 mg/kg/infusion heroin dosage in both protocols and the overall range of active lever presses and infusions was much broader for self-administration under this dosage. While the number of infusions, active lever presses and inactive lever presses over the last 3 days of acquisition did not differ significantly between the four experimental conditions (Kruskal–Wallis test, infusions: H(3) = 2.64, *p* = 0.451; active lever presses: H(3) = 0.20, *p* = 0.978; inactive lever presses: H(3) = 7.38, *p* = 0.061), animals in the 0.075 mg/kg dose groups administered a higher total amount of heroin, regardless of the type of cues paired with heroin infusion (Kruskal–Wallis test of total heroin intake: H (3) = 21.15, *p* < 0.0001; Dunn’s post-hoc tests: Exp 1, 0.075 mg/kg dose vs. 0.03 mg/kg dose, *p* = 0.003; Exp 1, 0.075 mg/kg dose vs. Exp 2, 0.03 mg/kg dose, *p* = 0.003; Exp 2, 0.075 mg/kg dose vs. 0.03 mg/kg dose, *p* = 0.017; Exp 1, 0.03 mg/kg dose vs. Exp 2, 0.075 mg/kg dose, *p* = 0.0193). To rule out differences in learning that may attribute to failure to acquire self-administration or variation in total number of infusions, we analyzed the number of lever presses during the time-out period, which occurred during the 20 seconds (s) immediately following a drug infusion, for all animals under both dosage and protocol conditions (Fig. 3A). Similar amounts of time-out pressing were observed for experiments 1 and 2, despite the differences in discrete and discriminative cues used during the two self-administration protocols. A significant main effect of time was observed but no time by dose and cue interactions were observed (two-way repeated measures ANOVA, main effect of time: *F*(9, 621) = 3.67, *p* = 0.0002; Fig. 3A).

Because animals in low dosage experiments had more time-out period exposure due to administration of a higher number of infusions, we calculated the number of lever presses during the time-out period divided by the number of infusions for each experimental protocol. Similarly, no time by dose and cue interactions were observed but a significant main effect of time was observed (Two-way Repeated measures ANOVA, main effect of time: *F*(9,621) = 3.77, *p* = 0.0002; Fig. 3B). These data indicated that differences in incubation of opioid craving in the four different experimental conditions cannot be attributed to failure to learn the acquisition protocol.

### Contribution of cues and heroin intake to incubation of opioid craving

**Figure 4:**
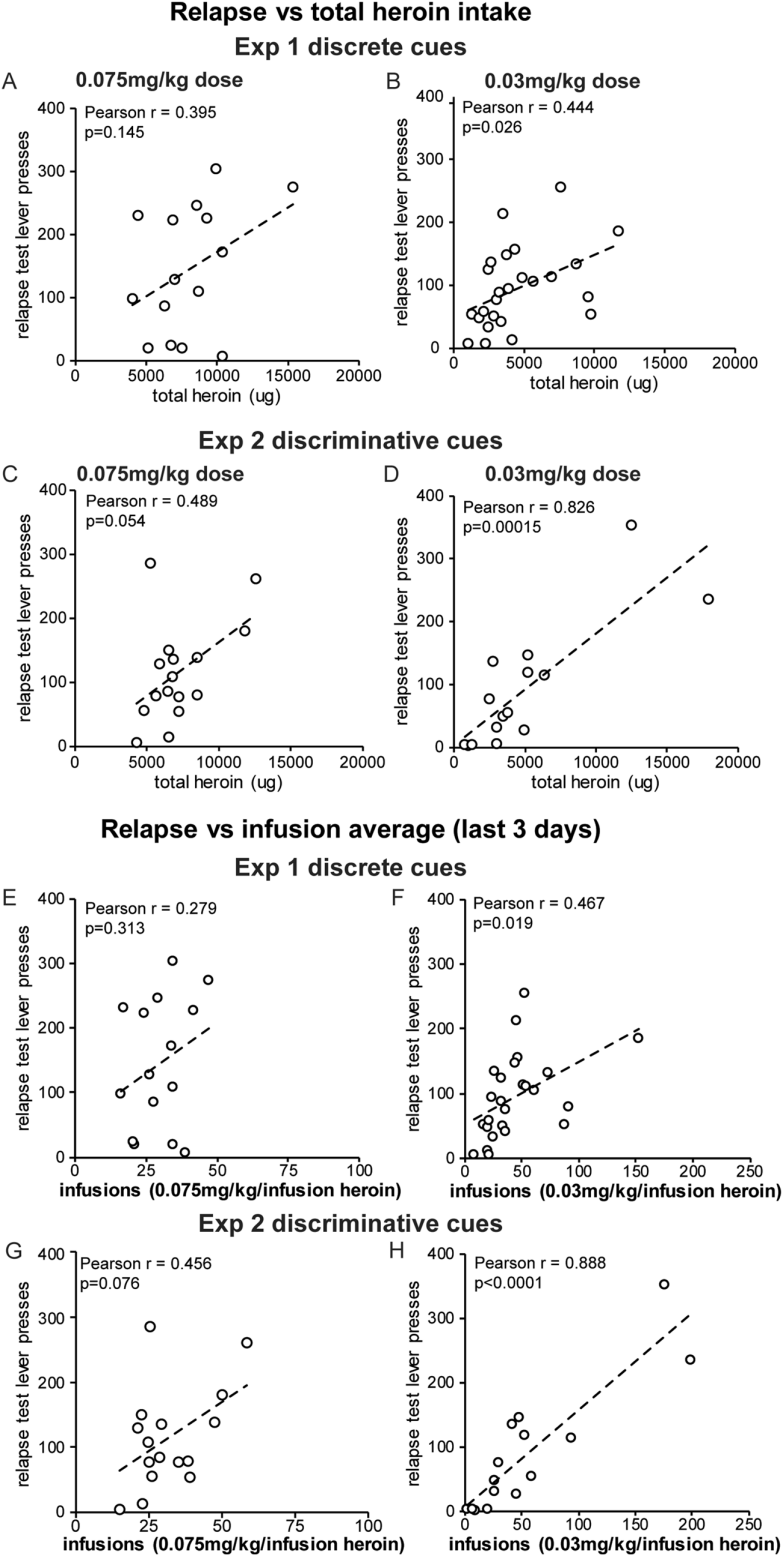
Total drug intake contributes to relapse behavior for low-dose heroin self-administration protocols with either discrete or discriminative drug cues. Lever pressing during the 90 min relapse test for both 2 and 21 days abstinence animals was correlated to each animal’s total drug intake during self-administration (**A**–**D**) or the average of infusions on the last three days of self-administration (**E**–**H**) for experiment 1 (**A**, **B**, **E**, **F**) and 2 (**C**, **D**, **G**, **H**). Pearson *r* values and *p*-values are shown for each comparison. *p < 0.05.

There is a range of self-administration behavior that exists for humans and preclinical rodent paradigms, with some animals self-administering more drug infusions than others. We examined the contribution of varied heroin intake to relapse for both heroin dosages in the discriminative or discrete drug cue protocols. First, we compared across protocols by examining the relationship between the number of infusions on the last 3 days of self-administration, as well as the heroin intake during self-administration, to relapse. Because there was no significant difference in active lever pressing for animals in Experiment 1 with the 0.03 mg/kg dosage or for Experiment 2 at any dosage, we examined whether the amount of lever pressing during the relapse test correlated to total drug intake or number of infusions administered, regardless of the length of abstinence. Total heroin intake and number of infusions were both significantly correlated to relapse lever pressing for the lower 0.03 mg/kg heroin dosage in both experiment 1 and 2. The correlation for the 0.075 mg/kg heroin dosage was not significant, although a strong trend was observed with discriminative cues in experiment 2 (Pearson correlation, 90 min relapse test vs total heroin intake: Experiment 1: 0.075 mg/kg, Pearson r = 0.395, *p* = 0.145; 0.03 mg/kg, Pearson r = 0.444, *p* = 0.026; Experiment 2: 0.075 mg/kg, Person r = 0.489, *p* = 0.054; 0.03 mg/kg, Pearson r = 0.826, *p* = 0.00015; 90 min relapse test vs average infusions: Experiment 1: 0.075 mg/kg, Pearson *r* = 0.279, *p* = 0.313; 0.03 mg/kg, Pearson *r* = 0.467, *p* = 0.019; Experiment 2: 0.075 mg/kg, Pearson *r* = 0.456, *p* = 0.076; 0.03 mg/kg, Pearson *r* = 0.888, *p* < 0.0001) (Fig. 4A–H). Correlation of average total active lever pressing during acquisition to relapse pressing resulted in similar values (Pearson correlation, 90 min relapse test vs average total active lever presses: Experiment 1: 0.075 mg/kg, Pearson *r* = 0.4207, *p* = 0.1770; 0.03 mg/kg, Pearson r = 0.3612, *p* = 0.076; Experiment 2: 0.075 mg/kg, Pearson r = 0.519, *p* = 0.0394; 0.03 mg/kg, Pearson r = 0.816, *p* = 0.0002). These results suggested that higher amounts of drug intake contributed to relapse behaviors when only discriminative cues were present, regardless of the drug infusion dosage. When discrete cues were presented with a low heroin dose, the total amount of drug self-administered promoted relapse and heavier use lead to higher more relapse behavior. Conversely, when discrete cues were presented in conjunction with a high dose of heroin, the total drug intake was irrelevant and drug craving increased in a time-dependent manner during abstinence.

We further explored this hypothesis to determine if the lack of incubation of opioid craving from discrete cues in the 0.03 mg/kg dosage was simply a result of less drug intake. To investigate this notion, we controlled for variation in heroin intake by analyzing the relapse behavior between animals that self-administered similar amounts of total heroin. We compared the relapse behavior for animals that self-administered higher amounts of total heroin during acquisition versus those that self-administer lower total amounts. We divided animals from each experiment and dosage into 4 groups: 2 days abstinence, high total heroin intake; 21 days abstinence, high total heroin intake; 2 days abstinence, low total heroin intake; 21 days abstinence, low total heroin intake. High heroin intake was defined as animals that self-administered more heroin than the median of all animals in each experimental protocol separately, while low heroin intake was defined as animals that self-administered less than the median of their respective experimental group.

**Figure 5:**
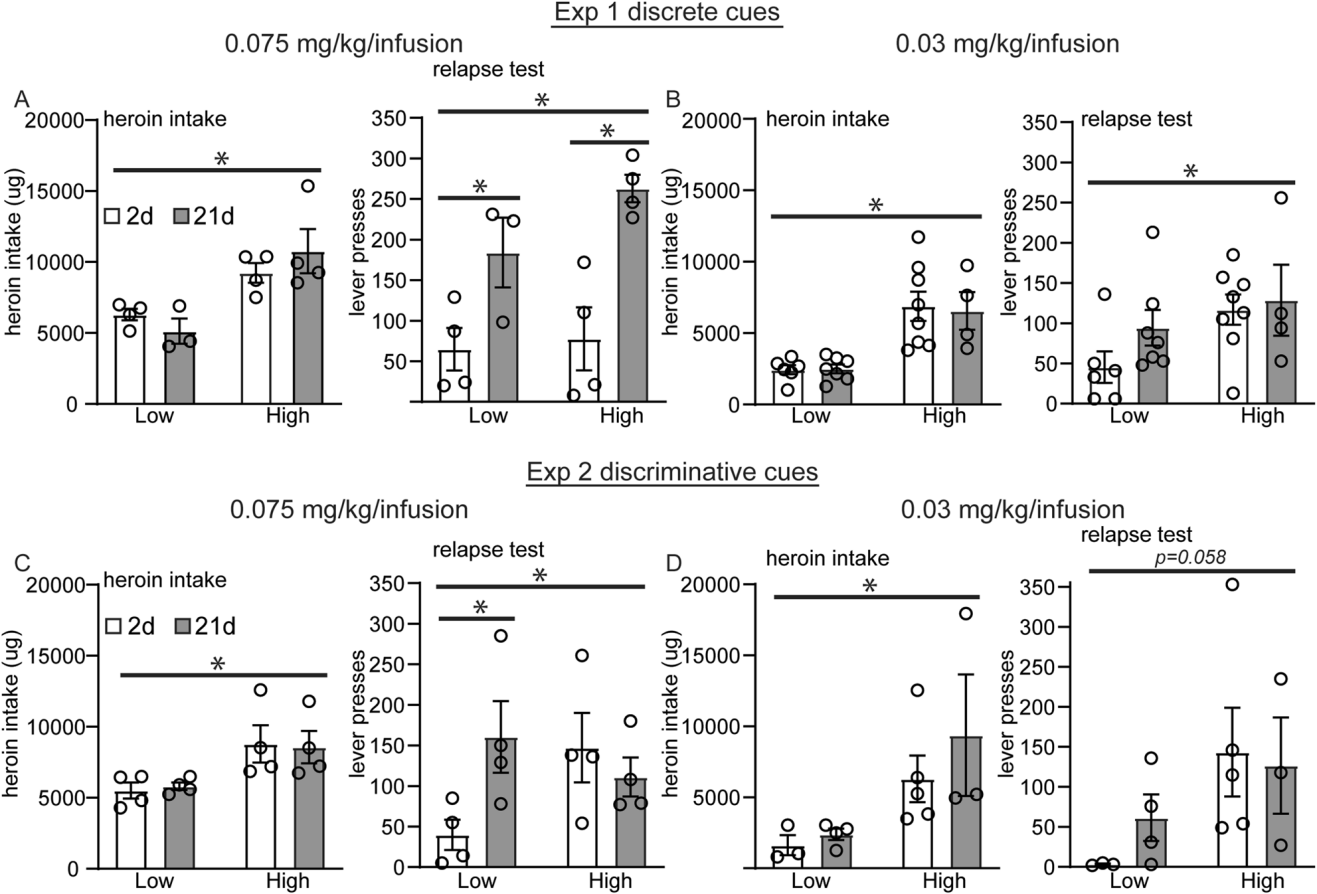
Variability in total drug intake contributes to incubation of craving for discriminative but not discrete drug cues at a 0.075 mg/kg/infusion dose. In each experimental protocol, some animals self-administered high total heroin intake or lower amounts of total heroin intake. Animals were classified as high or low heroin takers based on whether they were above or below the median total heroin intake for their experimental protocol. Shown are the average amounts of total heroin intake over 10 days of heroin self-administration as well as active lever pressing during a 90 min relapse test at 2 or 21 days after the last drug session for experiment 1 with discrete drug cues (**A**, **B**) and experiment 2 with discriminative drug cues (**C**, **D**). Error ± SEM. *p < 0.05.

Importantly, this method of analysis matched animals for drug intake at 2 and 21 days timepoints within each experimental condition (Fig. 5). In experiment 1, there was a significant main effect of abstinence on the relapse test in animals that self-administered the 0.075 mg/kg heroin dosage but no abstinence X heroin intake interaction was observed (two-way ANOVA, main effect of abstinence on relapse test: *F*(1,11) = 23.02, *p* = 0.0006; post hoc LSD test: high heroin 2 vs. 21 days, *p* = 0.001; low heroin 2 vs. 21 days, *p* = 0.027; Fig. 5A). Thus, total heroin intake had no relationship to lever pressing during the relapse test. Relapse was instead promoted by the amount of time an animal went through abstinence from this cue and dosage protocol. For animals that self-administered the 0.03 mg/kg heroin dosage with discrete cues in experiment 1, a significant main effect of heroin intake on relapse was observed but no abstinence × heroin intake interaction (Two-way ANOVA, main effect of heroin intake level on relapse test: *F*(1,21) = 4.59, *p* = 0.044; Fig. 5B). Animals that self-administered a low total amount of heroin over the course of the 0.03 mg/kg protocol tended to have higher lever pressing at 21 days of abstinence, but this result was not statistically significant.

In experiment 2, a significant abstinence X heroin intake interaction was observed in animals that self-administered the 0.075 mg/kg heroin dosage with discriminative cues but no main effects of heroin intake or abstinence day (Two-way ANOVA, abstinence × heroin intake interaction: *F*(1,12) = 5.25, *p* = 0.041; Fig. 5C). Animals that self-administered lower amounts of total heroin at this dosage and discriminative cue protocol had significantly more active lever presses at 21 days abstinence versus 2 days and displaying incubation behavior (post hoc LSD test: low heroin 2 vs. 21 days, *p* = 0.028; Fig. 5C). Lowering the heroin dosage to 0.03 mg/kg in combination with the discriminative drug cue protocol did not result in significant differences during the relapse test for high or low heroin takers, although low heroin takers displayed a trend similar to all other experimental groups.

## Discussion

The present study examined the contribution of drug dosage and drug associated cues to long lasting drug seeking behaviors. In a rat model of heroin self-administration, animals maintained a high motivation to seek heroin regardless of whether high (0.075 mg/kg/infusion) or low (0.03 mg/kg/infusion) doses of heroin were used. However, as previously described in the literature, heroin seeking was exacerbated after extended abstinence from a 0.075 mg/kg dose, leading to an incubation of craving^16,26^. In the operant learning paradigm that we have utilized, cues presented at the time of drug infusion allowed animals to form drug-cue associations that facilitated learning of the self-administration paradigm. We first replicated published findings that utilized a light and/or tone discrete cue to induce opioid craving after extended abstinence. Although most research groups use a light associated with the drug-contingent lever, there is not a uniform protocol that has been established for requirement of incubation of opioid craving. However, it has been shown that a compound stimulus (tone + light), instead of a singular stimulus itself, significantly increased cocaine seeking-behavior after extended abstinence, and this effect was more pronounced when the cocaine-related stimuli are presented in a contingent manner^28,29^.

We report that auditory and visual cues presented as conditioned stimuli for drug infusion supported robust incubation behavior at a high heroin dosage (0.075 mg/kg/infusion) but not at a low, previously untested dose (0.03 mg/kg/infusion). When the light cue was presented as a discriminative stimulus signaling drug availability in the absence of conditioned stimuli, heroin incubation behavior did not occur in all animals. These findings indicate that there were interactions between drug dosage and drug-associated cues that were required for time-dependent increases in opioid seeking after extended abstinence. This contrasted with a previous study of cocaine seeking behavior where a discriminative stimulus not paired to a conditioned stimulus was able to induce robust cocaine seeking behavior up to 300 days after abstinence^30^. Differences in the induction of incubation behavior from discriminative stimuli could be due to the class of drug self-administered. Indeed, heroin and cocaine elicit different behavioral profiles since they act on different physiological substrates^31^ and show different incubation profiles^32^. Session durations could have played a role too in failure of incubation: we used 6 h daily sessions, while Madangopal et al. used two 3 h daily sessions. Moreover, the experimental design used in the referenced cocaine study was different from the one we employed in experiment 2 because it trained animals to discriminate between a specific light stimulus coupled to the drug availability and a second stimulus light that was not coupled. Thus, the presentation of two light stimuli triggering different cause and effect relationships contributed to the associative learning. Discriminative stimuli paired with heroin availability were able to control drug-seeking only when presented briefly and contingently^31,33^. The presentation of the discriminative cue light above the active lever in experiment 2 might have failed to induce the conditioned response. Therefore, we hypothesize that short presentation of a light cue only during the infusion was more salient (experiment 1) than a light turning off during infusion (experiment 2). In addition, it is worth noting that the house-light turning off during the time-out period in experiment 1 has worked as occasion setter for the conditioned response together with the conditioned stimulus to better establish the associative learning^34^ required to enhance incubation behavior. On the contrary, in experiment 2, the house-light stayed on the entire session, even during drug-infusion. Discrete cues have been shown to be more potent than contextual in eliciting heroin-seeking behavior after abstinence^35,36^.

The fact that drug dosage was an essential factor for developing incubation of heroin seeking contrasted with an earlier study that examined the contribution of heroin self-administration dosage to contextual versus cue-induced relapse. In that study, Zhang et al. did not find differences in a relapse test between doses ranging from 0.025 to 0.1 mg/kg. However, the study also did not report incubation effects, likely due to the fact that the number of infusions was limited to 25 during acquisition^35^. It has been widely demonstrated that drug use and abuse is accompanied by the development of maladaptive drug-associated memories that contribute to relapse. Avoidance from abstinence symptoms, together with environmental cues, can recall associated memories previously formed as response^37,38^. Opioid abstinence symptoms are more severe than other drugs and play an important role in opioid relapse. However, low doses of opioids do not enhance memory^37^. Therefore, differences in the establishment of incubation at different doses could be due to the fact that high doses of a drug might elicit a stronger stimulation at the cellular level that ultimately might change the robustness of memory formation with respect to the lower dose. This does not explain why the 0.075 mg/kg dosage failed to induce incubation behavior when coupled with the discriminative protocol in high heroin takers. However, the answer might reside in the different processes that comprise the cue-associated learning processes. The tone and cue-light together with the dosage are the components responsible for perseverative drug-seeking behavior even in the absence of the drug-reinforcement. Several studies have reported that in reinstatement models of drug relapse, different types of brain areas and receptors contribute to heroin seeking behavior when the self-administration acquisition behavior is coupled with a discrete or discriminative cue^39^. Indeed, beside dopamine receptor activation, serotonin receptors (5-HT2A) are mostly implicated in discrete cues, while a cannabinoid receptor (CB1) was reported to be involved in discriminative cue-induced heroin reinstatement. Similarly, different brain areas are involved for the two paradigms: bed nucleus of the stria terminalis, ventral medial prefrontal cortex, pallidum and substantia nigra, basolateral amygdala and central amygdala for discrete cues; versus the medial prefrontal cortex, amygdala and nucleus accumbens core for discriminative cue. Reinstatement models and incubation models share the ability to precipitate relapse even in the absence of a reinforcing effect of the drug. Thus, it is possible to speculate that incubation behavior, as well, might be able to activate different kinds of receptors and brain areas when the learning acquisition behavior is led by a discrete or discriminative cue.

The lack of incubation with the lower 0.03 mg/kg dosage in both experiments 1 and 2 was not from animals failing to acquire self-administration behavior because the majority show a strong preference for the active versus inactive lever. Indeed, we determined that the discriminative stimulus used in experiment 2 did not induce differences in learning the self-administration paradigm as no differences in lever-pressing during the time-out period were observed. Surprisingly, the time-out pressing trends were similar among discrete and discriminative cue protocols at both high and low heroin dosages. Furthermore, animals that went through only 2 days of abstinence in each of the experiments had similar, strong levels of drug-seeking in the relapse test.

Deciphering which conditions may contribute to high or low levels of drug-seeking will advance our understanding of diverse populations of OUD patients. Because of this, we reported the range of drug intake and drug-seeking that occurs in each of the experiments with different dosages and drug cues. We reported that drug intake at the low 0.03 mg/kg heroin dose was the strongest predictor of relapse behavior at both 2 and 21 days of abstinence under both experimental conditions. Animals that self-administered the most drug at this dosage had the highest amount of drug-seeking behavior, regardless of the length of abstinence. While the effect was stronger when discriminative cues are used, we also detected significant correlations between relapse and total drug intake as well as number of infusions in experiment 1, when visual and auditory cues were presented as conditioned stimuli. Given that human opioid users experience a myriad of drug-associated cues in their environment which would be akin to experiment 2 with discriminative cues, this suggests that heavy opioid users, regardless of the length of usage, may have more persistent, long-lasting drug craving than light users. Indeed, a study of prescription opioid users demonstrated that those who used more prescribed opioids in the first month of usage were more likely to develop into long-term opioid users^40^.

In contrast, when the higher 0.075 mg/kg dosage of heroin was used together with discrete cues, the amount of drug infused during self-administration did not impact relapse. Instead, relapse was determined by the length of time of abstinence. When the higher dosage of heroin was coupled with discriminative cues, there was a significant correlation between active lever pressing and relapse, as well as a trend for a significant correlation between relapse and total heroin intake or average infusions. Animals that self-administered small amounts of the 0.075 mg/kg dosage, but not the 0.03 mg/kg dosage, displayed more drug seeking after 21 days of abstinence versus 2 days. Surprisingly, high total amounts of heroin infused at a dosage of 0.075 mg/kg with discriminative drug cues did not induce incubation. Indeed, lever pressing at 21 days of abstinence for the low heroin takers was similar to the animals with the same dosage but discrete cues. Together these data suggest that multiple mechanisms may contribute to perseverant drug seeking across a wide range of dose and cue interactions.

While we have not investigated neuroanatomical mechanisms of long-lasting heroin seeking as a function of drug dosage and cues here, the literature has elucidated a role for the corticolimbic system in the incubation of heroin craving^41^. Structures of the corticolimbic system activate immediate early genes during a relapse test after extended heroin abstinence^26,42^. Decreased cortical Fos activation correlates with blunted incubation of opioid craving in adolescent animals, relative to adults^42^. Thus, the lack of incubation observed when a lower 0.03 mg/kg heroin dose was employed could be attributed to a failure to engage the cortex. Finally, decreasing dopaminergic tone in the nucleus accumbens shell or the caudate putamen reduces morphine seeking in a relapse test after long-term abstinence^43^. Future studies that investigate the contribution of striatal dopamine to long-lasting heroin seeking may further delineate the molecular mechanisms responsible for varied responses in opioid relapse as a function of drug dosage and drug-associated cues. Such studies are critical to establish therapeutic targets for the treatment of OUD and maintenance of long-lasting abstinence from opioids.

## Methods

### Subjects

Seventy-three adult male Sprague Dawley rats (Charles River Laboratories), 7–8 weeks old, weighing 230–250 g upon arrival were pair-housed on a reverse 12- hour light/dark cycle (lights off at 9:00 A.M.) with constant room temperature (22 ± 2 °C) and humidity (40%). Animals were provided free access to laboratory chow and water. After 5–7 days of acclimation, animals underwent intravenous catheter surgery and were singly housed for the remainder of the study. All procedures followed the National Institutes of Health’s Guide for the Care and Use of Laboratory Animals and were approved by Temple University’s Institutional Animal Care and Use Committee. Experiments were performed in accordance with the ARRIVE Essential 10 Guidelines.

### Drug

Heroin hydrochloride was supplied by the National Institute on Drug Abuse drug supply program and was freshly dissolved in 0.9% sterile sodium chloride. Drug solutions were filtered by 0.2-micron syringe filter prior to use.

### Surgery

Rats were deeply anesthetized with isoflurane (3% induction, 2–2.5% maintenance) and were implanted with silastic tubing catheters (SAI Infusion Technologies, Lake Villa, IL, USA) in the right jugular vein under aseptic conditions. The catheter was fixed to a 22G stainless steel cannula cemented with a polypropylene mesh backmount (PlasticsOne Technologies, Roanoke, VA, USA) and passed subcutaneously over the shoulder, exiting in the mid-scapular region. Animals were given 5–7 days for recovery following surgery. During recovery, rats were monitored daily for weight and behavior changes and received meloxicam (1 mg/kg, injected subcutaneously) and antibiotics (Cefazolin 10 mg/kg, injected intravenous). Catheters were flushed with heparinized saline solution (10 USP/ml) every day during recovery and self-administration. The day before starting self-administration, animals were tested for catheter patency with propofol (1%) and food restricted to standard rat chow that measured 10% of their bodyweight. Food-restriction during self-administration did not impair their performance or decrease their bodyweight (Supplemental Fig. 1) and was used to increase motivation to perform the task. Immediately after the 10th heroin self-administration session, animals were given food ad libitum for the remainder of the study. Thus, no animals were food-restricted during the relapse test as food deprivation can increase heroin reinstatement^44^.

### Equipment

Drug self-administration studies were conducted in operant chambers (29.5 × 32.5 × 23.5 cm, Med Associates, Fairfax, VT, USA) encased in a sound-attenuated and ventilated cubicle (63.5 × 60.96 × 42.55 cm). Chambers were equipped with two retractable levers positioned 12 cm apart, 8 cm from the grid and extending 1.5 cm into the box. A cue light stimulus was located above levers and a cue house-light was positioned on the opposite wall. An acoustic cue compartment was located next to the house light wall. Drug infusions were delivered by a syringe pump (Med Associates, Fairfax, VT, USA) through a stainless-steel single-channel swivel (Instech Laboratories, Inc., Plymouth Meeting, PA, USA) and polyethylene tubing encased in a metal spring connecting the swivel to the catheter fitting on the animal's back.

### Procedure

Self-administration procedures were conducted under a fixed ratio (FR) 1 schedule of reinforcement where every active lever press resulted in a drug infusion (0.1 ml/kg/infusion) delivered at a rate of 17.86 µl per second. Animals were trained for 10 days of acquisition in 6-h daily sessions. After that, rats were split into two groups and underwent forced abstinence for 2 or 21 days in their home cage. To control for differences in drug self-administration, treatment groups were not randomized. Researchers balanced each experimental group for a given dosage and protocol to ensure that lever pressing and infusions were equivalent between animals that underwent 2 or 21 days of abstinence and were therefore not blinded during the relapse test. After each time-point limit, animals were re-exposed to the self-administration chambers for a 90-min relapse test with the same protocol employed in acquisition. All of the drug-associated cues present during acquisition were presented in the same manner during the relapse when the animal pressed the active lever. However, active lever pressing did not result in any drug infusion. To separate animals into high or low heroin takers, we defined high heroin intake as self-administration of more heroin than the median of all animals in each experimental protocol separately, while low heroin intake was defined as animals that self-administered less than the median of their respective experimental group, as previously described^45–47^.

#### Experiment 1

When the animal began the session, the house light became illuminated and both the active and inactive levers extended. Active lever pressing resulted in (a) illumination of the stimulus light above the active lever during infusion; (b) presentation of the acoustic cue (2.9 kHz, 65 dB) for 2 s; and (c) activation of the infusion pump for intravenous infusion of drug solution (Fig. 1). Each infusion was followed by a 20 s time-out period, during which the house light was turned off and the active lever was not responsive. Both levers remained extended during the time-out. After the time-out period, the house light became illuminated again, signaling the availability of drug. Lever pressing was recorded during the entire session, including during the time-out period. The inactive lever was not coupled to any effect. To evaluate the contribution of heroin dosage to time-dependent drug-seeking behaviors, two different doses were tested in this context: a dose of 0.075 mg/kg/infusion (n = 16; Fig. 1B–D) and a lower dose of 0.030 mg/kg/infusion (n = 25; Fig. 1E–G). One animal died after the conclusion of self-administration, ~ 1–2 weeks into incubation, and was excluded from the relapse test.

#### Experiment 2

To evaluate the role of discriminative cues in the expression of the incubation of craving, we manipulated the light and tone cues delivered during self-administer heroin. The stimulus light cue worked as a discriminative signal of drug availability (Fig. 2). When the animal began the session, the house light and the cue light over the active lever were illuminated. The house light remained illuminated during the entire session. Active lever pressing resulted in (a) the stimulus light above the active lever turned off; and (b) activation of the infusion pump for intravenous infusion of drug solution. Each infusion was followed by a 20 s time-out period, during which the light above the active lever was not illuminated and the active lever was not responsive. Both levers remained extended during the time-out. After the time-out period, the light above the active lever became illuminated again, signaling the availability of drug. There were no auditory cues in this protocol and the inactive lever was not coupled to any effect. Similar to experiment 1, two different doses of heroin were tested in this context: a dose of 0.075 mg/kg/infusion (n = 16; Fig. 2B–D) and lower dose of 0.030 mg/kg/infusion (n = 16; Fig. 2E–G). The high dosage has been well established in the literature to induce incubation of heroin craving with a discrete drug cue protocol and thus served as the positive control. One animal died after the conclusion of self-administration, ~ 1–2 weeks into incubation, and was excluded from the relapse test.

### Statistical analysis

All data are presented as mean ± standard error of the mean (SEM). Two-way repeated measures analysis of variance (ANOVAs) were used to analyze acquisition data over 10 days. Unpaired student’s *t *tests were used to analyze the cumulative 90-min relapse test for each experiment separately. Two-way ANOVAs with LSD post hoc tests were used to compare heroin intake and abstinence day interactions in the two experimental protocols. D’agostino and Pearson normality tests were performed to determine if data was normally distributed. To compare the number of infusions and lever presses across the two experimental protocols, Kruskal–Wallis tests with Dunn’s post hoc tests were performed. Pearson correlations were performed to compare total heroin intake to lever pressing. A *p* value of less than 0.05 (*p* < 0.05) was considered statistically significant. All analyses were performed using the GraphPad software package (Prism version 8; GraphPad, San Diego, CA, USA).

### Supplementary Information

Supplementary Figures.
