# Author Correction: Drug-associated cues and drug dosage contribute to increased opioid seeking after abstinence

**DOI:** 10.1038/s41598-021-03383-9

**Published:** 2021-12-07

**Authors:** Mary Tresa Zanda, Gabriele Floris, Stephanie E. Daws

**Affiliations:** 1 Center for Substance Abuse Research, Temple University, 3500 N Broad St, MERB/Rm 847, Philadelphia, PA 19140 USA; 2 Department of Neural Sciences, Temple University, Philadelphia, PA USA

Correction to: *Scientific Reports* 10.1038/s41598-021-94214-4, published online 21 July 2021

The original version of this Article contained errors in Figures 1 and 2, where the figure legends in panels D and G, “Relapse test”, incorrectly showed a black and a grey square to denote the two treatment groups. The correct histogram colour for the “21D” and “2D” groups is grey and white, respectively.

Furthermore, Figure 5 contained an error in panel A, where the histogram for the “21D low” group was reversed with the histogram for the “2D high” group inside the “heroin intake” graph.

The original Figures 1, 2 and 5 and accompanying legends appear below.

**Figure 1:**
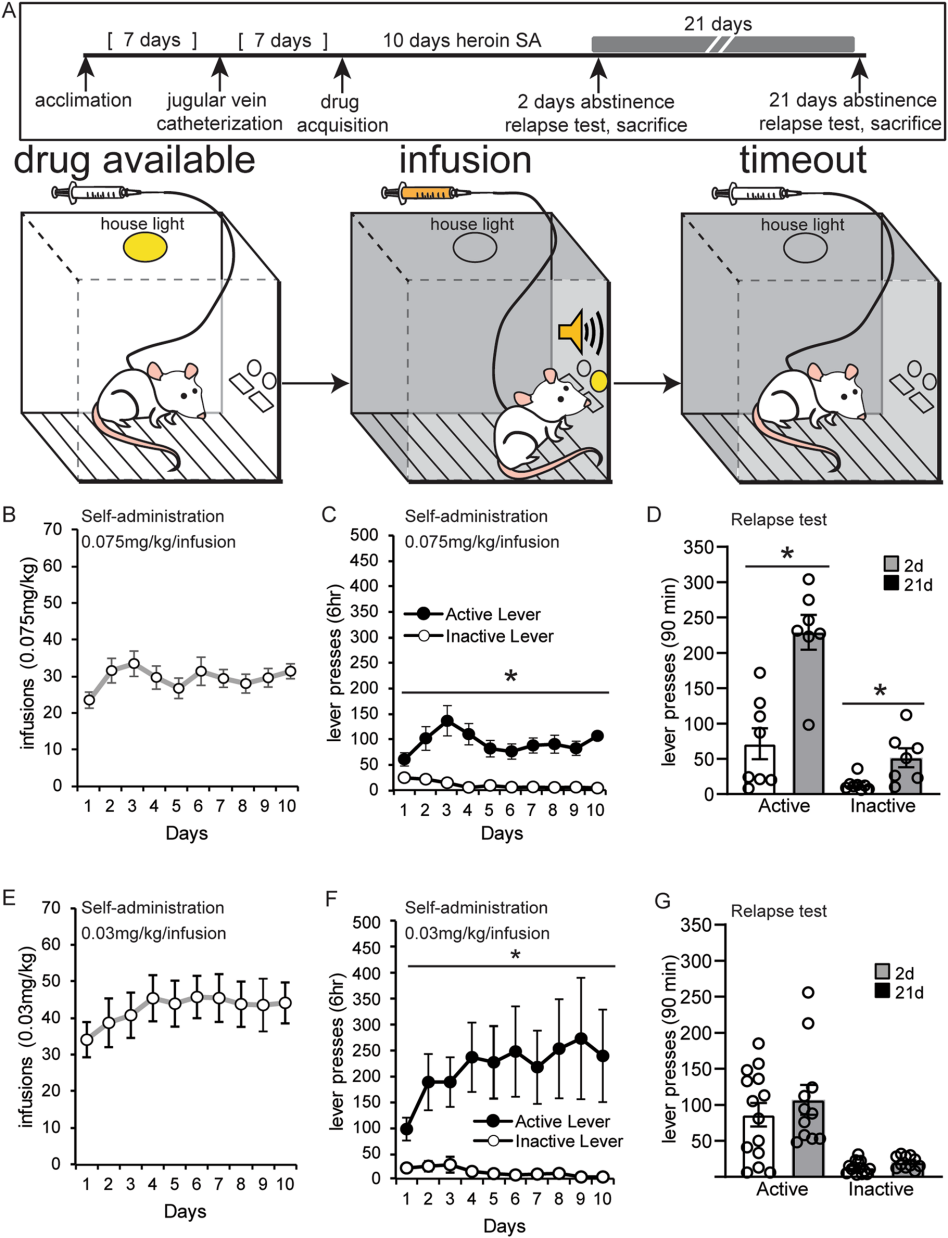
Establishment of a rat incubation of opioid craving protocol with discrete drug cues. (**A**) An overview of the protocol to measure drug seeking after abstinence from self-administration of heroin paired with discrete drug cues. Animals underwent surgery for jugular vein catheterization then acquired heroin self-administration in 6-h daily sessions for 10 days. A relapse test was performed after 2 or 21 days of abstinence from heroin. During acquisition, the house light signaled the availability of the drug. Active lever pressing resulted in illumination of a light above the active lever and an auditory cue during drug infusions. A 20 s (s) timeout period followed each infusion, in which no lights were illuminated in the chamber. (**B**, **C**, **E**, **F**) The number of infusions and lever presses during heroin self-administration at a 0.075 mg/kg/infusion dosage (**B**, **C**) and a lower 0.03 mg/kg/infusion dosage (**E**, **F**). Error ± standard error of the mean (SEM). (**D**, **G**) Lever pressing during the relapse test after forced abstinence from the 0.075 mg/kg/infusion dosage (**D**) and the 0.03 mg/kg/infusion dosage (**G**). *p < 0.05.

**Figure 2:**
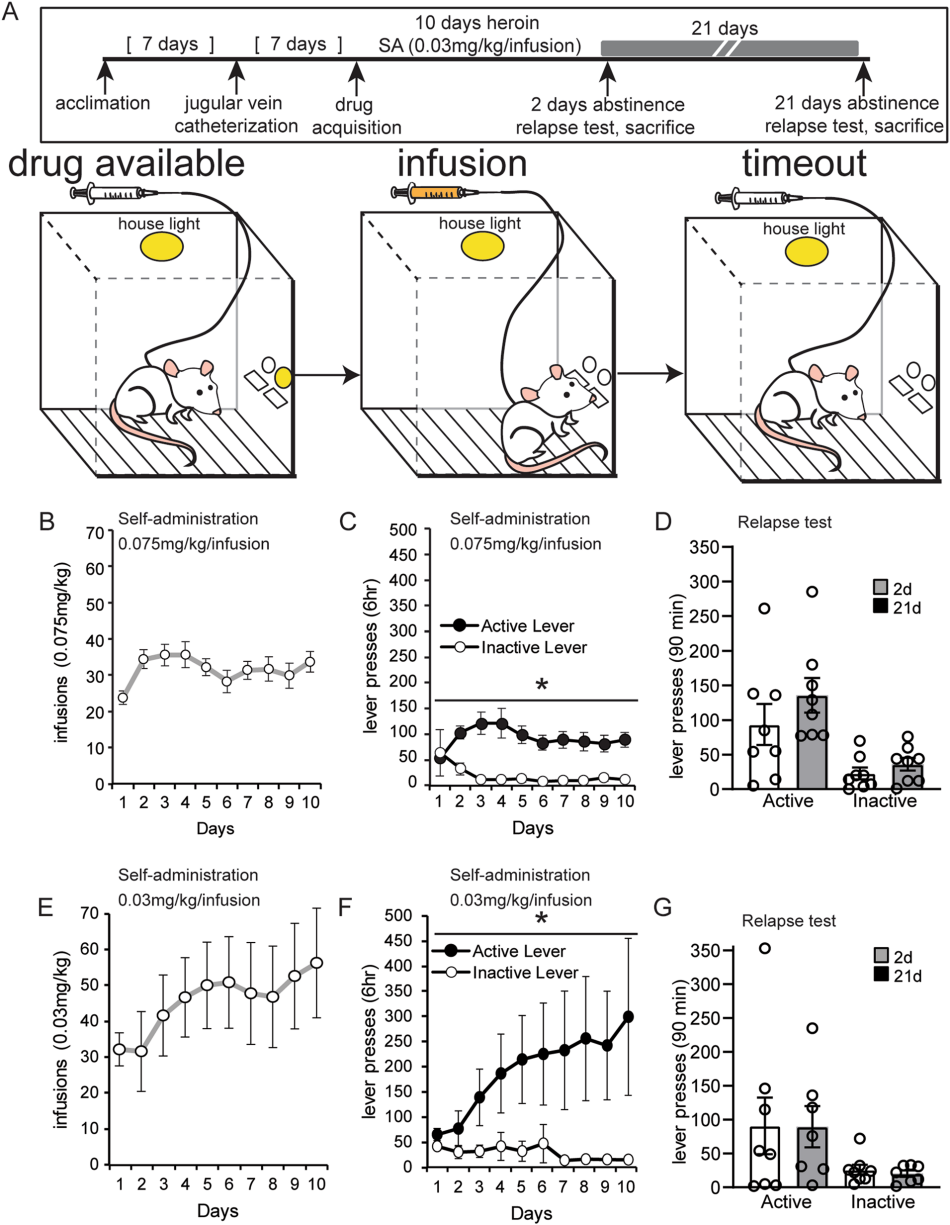
Discriminative cues produce steady drug-seeking during abstinence. (**A**) An overview of the protocol to measure drug seeking after abstinence from heroin self-administration using discriminative drug cues. Animals underwent an identical experimental protocol as described in Experiment 1 except with modified light and tone cues. (**B**, **C**, **E**, **F**) The number of infusions and lever presses during heroin self-administration at a 0.075 mg/kg/infusion dosage (**B**, **C**) and a lower 0.03 mg/kg/infusion dosage (**E**, **F**). (**D**, **G**) Lever pressing during the relapse test after forced abstinence from the 0.075 mg/kg/infusion dosage (**D**) and the 0.03 mg/kg/infusion dosage (**G**). Error ± SEM. *p < 0.05.

**Figure 5:**
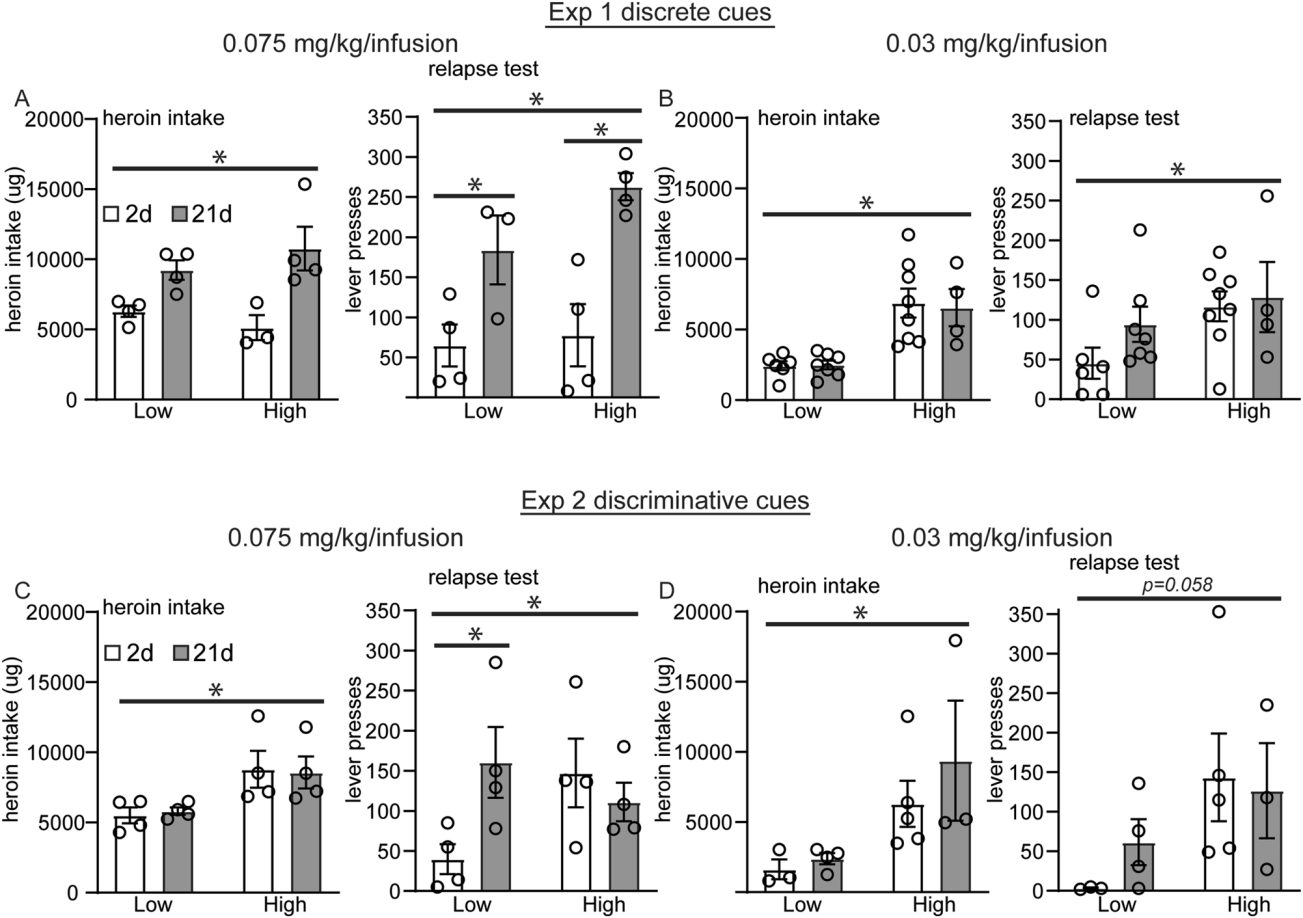
Variability in total drug intake contributes to incubation of craving for discriminative but not discrete drug cues at a 0.075 mg/kg/infusion dose. In each experimental protocol, some animals self-administered high total heroin intake or lower amounts of total heroin intake. Animals were classified as high or low heroin takers based on whether they were above or below the median total heroin intake for their experimental protocol. Shown are the average amounts of total heroin intake over 10 days of heroin self-administration as well as active lever pressing during a 90 min relapse test at 2 or 21 days after the last drug session for experiment 1 with discrete drug cues (**A**, **B**) and experiment 2 with discriminative drug cues (**C**, **D**). Error ± SEM. *p < 0.05.

The original Article has been corrected.

