# Supplementary material for: Drug-associated cues and drug dosage contribute to increased opioid seeking after abstinence
Source: Sci Rep. 2021 Jul 21;11:14825. doi: 10.1038/s41598-021-94214-4 (PMC8295307; doi:10.1038/s41598-021-94214-4)

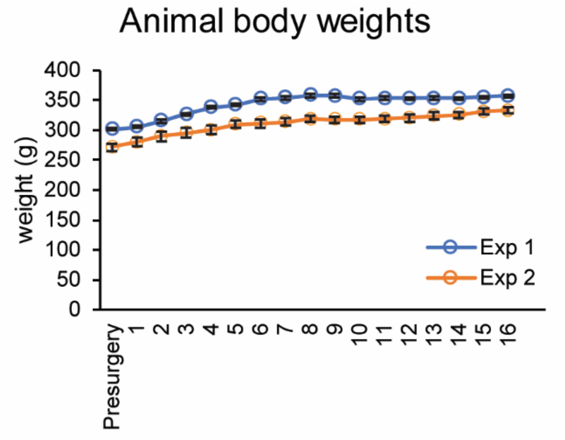

Supplemental Figure 1: Stable maintenance of weight during heroin self-administration. Rats were weighed daily before jugular vein catheterization surgery, during surgery recovery and throughout heroin self-administration. Shown are the average rat weights in grams (g) for each experiment. Error = +/- standard error of the mean (SEM).

Supplemental Figure 2: Lever pressing during each 30 minute bin of the 90 minute relapse test for animals that previously self-administered the 0.03mg/kg/infusion heroin dosage with discrete drug cues. Error +/- SEM. *p<0.05.

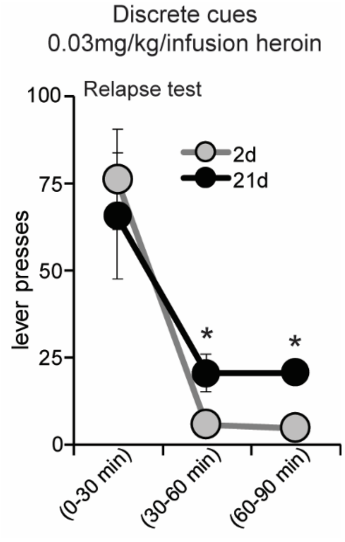

Supplemental Figure 3: Relapse test data for animals that previously self-administered the 0.03mg/kg/infusion dosage with discrete (A) or discriminative (B) cues and met acquisition criteria. Error = +/- standard error of the mean (SEM).

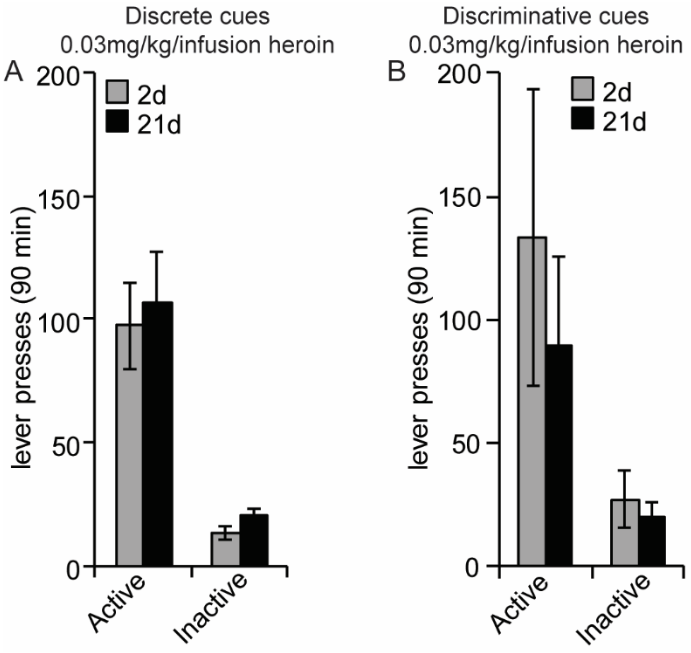

Supplement: Supplementary file 1 — Supplementary Figures. [file 41598_2021_94214_MOESM1_ESM.pdf]
